# Disseminated Metastatic Tubulopapillary Mammary Carcinoma in a Captive Fennec Fox (*Vulpes zerda*)

**DOI:** 10.1002/vms3.71051

**Published:** 2026-07-02

**Authors:** Byungkwan Oh, Chae Woong Lim, Sang‐Ik Oh, Bumseok Kim

**Affiliations:** ^1^ Biosafety Research Institute and College of Veterinary Medicine Jeonbuk National University Iksan Republic of Korea

**Keywords:** estrogen receptor alpha, fennec fox, mammary gland carcinoma, metastasis, tubulopapillary carcinoma

## Abstract

**Background:**

Mammary gland tumours (MGTs) are common in women and domestic carnivores but appear to be rare in wild canids.

**Case presentation:**

A 14‐year‐old female fennec fox (*Vulpes zerda*) was hospitalised due to dyspnea, received supportive treatment, but died 3 days later and was submitted for necropsy. Gross examination revealed an emaciated fox with multiple firm white nodules involving the mammary gland region, left pectoral muscle, lungs and pleural surfaces. Histologically, these nodules consisted of an infiltrative mammary epithelial neoplasm forming tubulopapillary structures with marked nuclear pleomorphism, characterised by hyperchromatic, clumped nuclei. Immunofluorescence labelling revealed strong estrogen receptor alpha (ERα) expression confined to the cytoplasm of tumour cells across all examined sites, an unusual pattern, compared with the typically nuclear ERα staining reported in MGTs.

**Conclusions:**

Based on these findings, the MGT in this fennec fox was diagnosed as a Grade II tubulopapillary carcinoma. This case represents the first description of a mammary gland carcinoma with widespread systemic metastasis in a fennec fox.

## Graphical abstract

A captive female fennec fox developed a disseminated Grade II tubulopapillary mammary carcinoma with metastases to the mammary gland region, pectoral muscle, lungs and pleural cavity. Tumour cells at all sites showed strong cytoplasmic, rather than nuclear, estrogen receptor alpha immunofluorescence. This case represents the first description of a mammary carcinoma with widespread systemic metastasis and aberrant cytoplasmic ERα expression in a fennec fox.

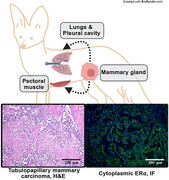

1

Mammary gland tumours (MGTs) are among the most common neoplasms in humans, dogs and cats (Goldschmidt et al. 2016). In female dogs, MGTs accounted for 41.7% of all cancers in a large registry‐based study (Dorn et al. 1968). Approximately half of canine MGTs are histologically malignant, and many are high‐grade carcinomas with a high risk of metastasis to regional lymph nodes and the lungs (Rasotto et al. 2012; Zheng et al. 2022). Among malignant subtypes, tubulopapillary carcinoma is one of the most frequent histological forms and is associated with a substantial risk of tumour‐related death (Rasotto et al. 2017). Overall, malignant MGTs carry high mortality if untreated, and advanced or metastatic disease, particularly involving regional lymph nodes or distant organs, is a strong predictor of poor prognosis (Moon et al. 2022; de Araújo et al. 2015). Unlike their high prevalence in dogs and cats, MGTs appear to be uncommon in most other mammalian species and have only rarely been documented in zoo‐housed and free‐ranging wild animals (Munson and Moresco 2007).

The fennec fox (*Vulpes zerda*) is the smallest canid species and is adapted to desert environments in North Africa (Kang et al. 2021). This nocturnal fox is commonly maintained in zoological collections. In a 40‐year retrospective review of managed fennec foxes, the mean age at death of adult individuals was approximately 9.8 years (Figueroa et al. 2023). In captive populations, neoplastic diseases have been reported only infrequently and is dominated by hepatocellular neoplasms, with a few other tumour types. To date, however, MGTs have not been reported in this species (Monahan et al. 2018; Shekelle et al. 2025).

A 14‐year‐old intact female fennec fox was hospitalised after sustaining multiple bite wounds to the tail and perineal region inflicted by cage mates during the breeding season. The animal showed marked tachypnea and episodic open‐mouth breathing but maintained normal appetite and activity in the enclosure. Supportive treatment, including oxygen supplementation, antibiotics, anti‐inflammatory analgesics and parenteral nutritional support, was provided; however, clinical examination and diagnostic imaging were not performed because the fox became extremely agitated and developed severe hyperpnea during restraint. The fox died suddenly on the third day of hospitalisation and was submitted for a necropsy.

Grossly, the fox was moderately emaciated and weighed 0.97 kg (Figure 1A). A large volume of clear, serous pleural effusion was present within the thoracic cavity (Figure 1B). Multiple firm, white nodules measuring up to approximately 1 × 2 cm were present in several mammary glands along the mammary chain and were also observed within the left pectoral muscle, lungs, pericardium, parietal pleura and thoracic wall (Figure 1C–F).

**FIGURE 1:**
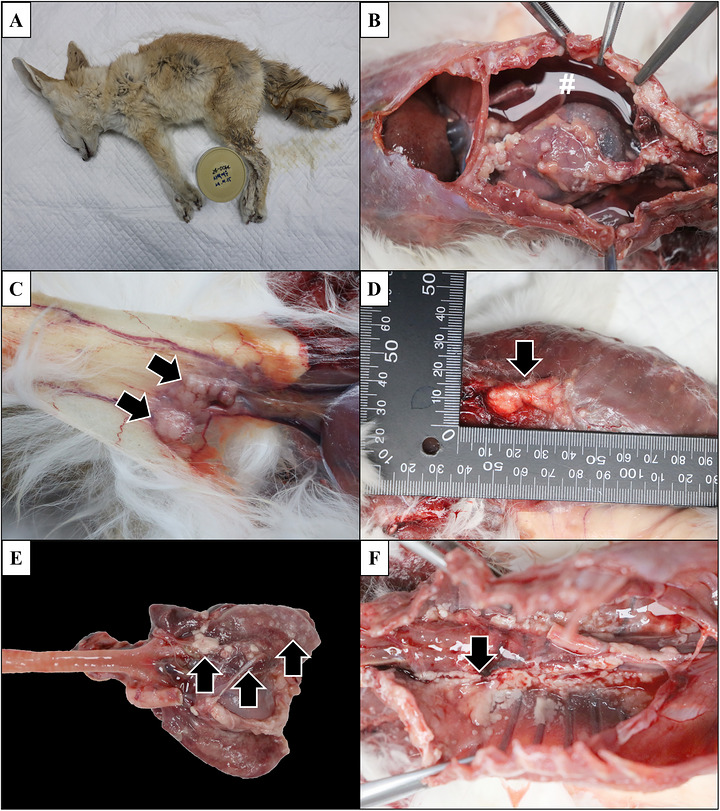
Representative gross lesions of a mammary gland carcinoma and metastases in a fennec fox. (A) The fox was moderately emaciated. (B) A large volume of clear pleural effusion (hash mark) was present within the thoracic cavity. Multifocal firm white nodules (arrows) were observed in (C) the mammary gland region, (D) the left pectoral muscle, (E) the lungs, and (F) the parietal pleura and the thoracic wall.

For histopathology, collected tissues were fixed in 10% neutral buffered formalin, routinely processed, embedded in paraffin wax and sectioned at 4 µm. Sections were stained with haematoxylin and eosin (H&E) using an automated stainer. For immunofluorescence, sections were incubated with rabbit anti‐estrogen receptor alpha (ERα) antibody (ab3575, Abcam, Cambridge, UK), followed by Alexa Fluor 488‐conjugated chicken anti‐rabbit IgG (Invitrogen, Waltham, MA, USA) as the secondary antibody. Nuclei were counterstained with DAPI (H‐1200; Vector Laboratories, Burlingame, CA, USA) and mounted with VECTASHIELD Antifade Mounting Medium. This study did not involve the use of any cell lines.

Histopathologically, the nodules were composed of proliferative mammary epithelial cells forming tubulopapillary structures (Figure 2A). The neoplastic cells exhibited marked nuclear pleomorphism with hyperchromatic nuclei, clumped chromatin and mitotic figures were observed (15 per 10 high power field [HPF] at 400× magnification; Figure 2B). The lumina of many neoplastic tubules and solid nests contained eosinophilic proteinaceous material. A severe infiltrate of mononuclear inflammatory cells surrounded the neoplastic mammary epithelium (Figure 2C). Lesions with identical histomorphology were also present in the left pectoral muscle, lungs and thoracic wall (Figure 2D–F). To confirm that the neoplasm at these extra‐mammary sites was of mammary origin, immunofluorescence staining for ERα, a representative marker of mammary epithelium, was performed. Immunofluorescence revealed strong cytoplasmic positivity for ERα in the neoplastic cells of the mammary gland, and an identical cytoplasmic staining pattern was observed in neoplastic cells within the thoracic wall and lungs (Figure 3). On the basis of these histopathologic features, the mammary mass was diagnosed as a Grade II tubulopapillary carcinoma, and metastatic lesions with the same histomorphology were identified in the left pectoral muscle, lungs, pericardium, parietal pleura and thoracic wall.

**FIGURE 2:**
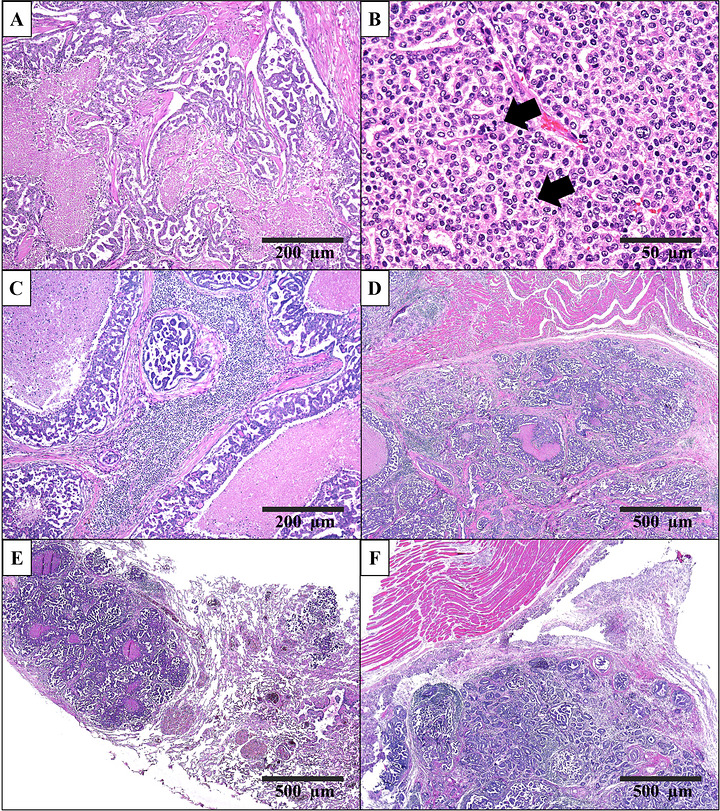
Representative histologic features of a tubulopapillary mammary carcinoma and metastatic lesions in a fennec fox. (A) Neoplastic mammary epithelial cells form distinct tubulopapillary structures in the mammary gland (haematoxylin and eosin [H&E], ×100). (B) Neoplastic cells exhibit marked nuclear pleomorphism, characterised by hyperchromatic nuclei with clumped chromatin and frequent mitotic figures (arrows) in the mammary gland (H&E, ×400). (C) A severe infiltrate of mononuclear inflammatory cells surrounds the neoplastic mammary epithelium (H&E, ×100). Metastatic lesions with identical histomorphology infiltrate (D) the pectoral muscle, (E) the lungs, and (F) thoracic wall (H&E, ×40).

**FIGURE 3:**
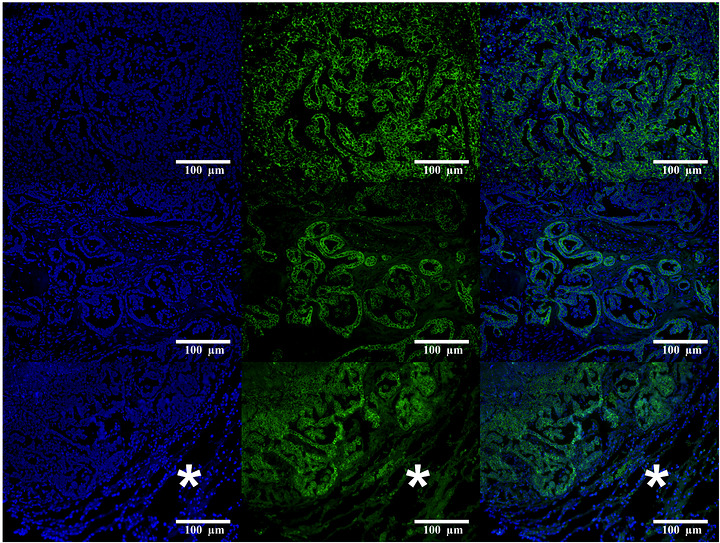
Representative immunofluorescence labelling for estrogen receptor alpha (ERα) in a tubulopapillary mammary carcinoma and metastases in a fennec fox. Strong cytoplasmic ERα immunoreactivity (FITC, green) is present in neoplastic epithelial cells in the primary mammary tumour, thoracic wall metastasis, and pulmonary metastasis. Adjacent nonneoplastic pulmonary parenchyma (asterisk) shows no ERα labelling, with nuclei counterstained with DAPI (blue; ×200).

Spontaneous MGTs are rarely reported in nondomestic canids and other wildlife, in contrast to their high prevalence in domestic dogs and cats. In a comparative review of MGTs in wildlife, no tumours were detected among intensively monitored populations of free‐ranging cheetahs (*Acinonyx jubatus*) and island foxes (*Urocyon littoralis*), despite more than 500 necropsies performed (Munson and Moresco 2007). Among wild canids, only a few cases of MGTs have been described, including mammary carcinomas in a Mexican gray wolf (*Canis lupus baileyi*), a red fox (*Vulpes vulpes*), and a maned wolf (*Chrysocyon brachyurus*; Gamba et al. 2011). The close histologic similarity between the present case and canine MGTs suggests that grading systems established for domestic dogs can be applied to assess the malignancy of MGTs in nondomestic canids (Goldschmidt et al. 2011). This supports the use of comparative pathological approaches to predict the biological behavior of rare MGTs in wildlife, for which species‐specific criteria are currently lacking. In the canine grading system, mitotic counts of 10–19 per 10 HPF receive an intermediate score, whereas ≥ 20 mitoses/10 HPF receive the highest mitotic activity score; the mitotic count in this case (15/10 HPF) fell within the intermediate range (Goldschmidt et al. 2016).

In a previously reported maned wolf case, a tubulopapillary mammary carcinoma was confined to the mammary gland and showed lymphatic and stromal invasion but no documented distant metastases (Gamba et al. 2011). In contrast, the fennec fox in the present report had a tubulopapillary mammary carcinoma with widespread metastases not only to the lungs, a common target organ for metastatic canine MGTs, but also to the pectoral muscle, pericardium, parietal pleura and thoracic wall. This pattern is consistent with a more recent description of a metastatic Grade II simple tubulopapillary mammary carcinoma in a free‐living maned wolf (Oliveira Junior et al. 2023), which also exhibited metastases to regional lymph nodes, lungs, parietal pleura and pericardium. Taken together, these cases demonstrate that MGTs in wild canids can exhibit highly aggressive biological behavior comparable to that seen in domestic dogs, challenging earlier suggestions of limited metastatic potential.

Furthermore, fennec foxes in zoological collections often experience increased longevity compared to their free‐ranging counterparts and may therefore be at increased risk for age‐related neoplastic diseases (Terio et al. 2018). In domestic dogs and cats, prolonged exposure to endogenous estrogen and progesterone in intact females is a well‐established risk factor for mammary carcinomas, and early ovariohysterectomy substantially reduces this risk (Torres et al. 2021, Overley et al. 2005). The fennec fox in this report was an intact 14‐year‐old female, considerably older than the reported mean lifespan in captivity (Figueroa et al. 2023); it is therefore plausible that long‐term cyclical hormonal stimulation associated with repeated breeding seasons contributed to the development of this neoplasm. This report highlights the importance of incorporating regular mammary gland palpation and appropriate diagnostic imaging into geriatric health screening protocols for fennec foxes and other zoo‐housed nondomestic canids.

ER is one of the principal biomarkers used to characterise MGTs and to predict their biological behavior in both women and dogs (Nieto et al. 2000; Ali and Coombes 2000). Although ER status is typically assessed on the basis of nuclear immunoreactivity, rare examples of predominantly cytoplasmic ERα staining have been documented in human breast carcinomas (incidence < 2%) and have been associated with expression of abnormal ER isoforms, including ER‐α36 and ER46, which exhibit cytoplasmic or membranous localisation and altered nongenomic signalling functions (Welsh et al. 2012). In canine MGTs, ERα immunolabelling has generally been described as nuclear, when present, and Erα expression is more frequently detected in benign and low‐grade lesions than in high‐grade carcinomas (Nieto et al. 2000). In contrast, the present case showed strong ERα immunoreactivity confined to the cytoplasm of neoplastic cells in the mammary gland, thoracic wall and lungs despite its high histologic grade and extensive metastatic spread, representing an unusual hormone receptor profile. Similar mechanisms involving abnormal ER isoforms, as described in human breast cancer, may underlie the cytoplasmic localisation observed in this case. Whether this pattern reflects species‐specific features of MGTs in fennec foxes or individual variation remains unclear, and additional cases will be required to clarify its significance.

Consistent with this uncertainty, only ERα could be evaluated by immunofluorescence in this case, and additional hormone receptors such as progesterone were not assessed. Consequently, the molecular subtype of the carcinoma remains incompletely defined. In addition, severe agitation and hyperpnea during restraint prevented safe antemortem imaging and blood sampling, which restricts the clinicopathologic correlations that can be made in this case.

To the best of the authors’ knowledge, this is the first report of a malignant MGT in a fennec fox with disseminated metastases to the lungs, pectoral muscle, pericardium, parietal pleura and thoracic wall. The extensive metastatic dissemination observed in this case indicates that tubulopapillary mammary carcinoma in fennec foxes can exhibit aggressive biological behavior comparable to that described for canine MGTs. This report may provide useful reference information for the diagnosis, prognostic assessment and clinical management of MGTs in fennec foxes and other nondomestic canids.

## Author Contributions

**Byungkwan Oh**: conceptualisation, methodology, investigation, data curation, visualisation, and writing – original draft. **Byungkwan Oh, Sang‐Ik Oh**, **Bumseok Kim**: validation. **Chae Woong Lim, Sang‐Ik Oh, Bumseok Kim**: supervision. **Bumseok Kim**: funding acquisition, project administration and resources. **Byungkwan Oh, Bumseok Kim**: Writing – review and editing.

## Conflicts of Interest

The authors declare no conflicts of interest.

## Data Availability

The data that support the findings of this study are available from the corresponding author upon reasonable request.
